# The Effect of Curcumin on Human Bronchial Epithelial Cells Exposed to Fine Particulate Matter: A Predictive Analysis

**DOI:** 10.3390/molecules171012406

**Published:** 2012-10-22

**Authors:** Zhiguo Zhang, Xuyan Niu, Cheng Lu, Miao Jiang, Gary G. Xiao, Aiping Lu

**Affiliations:** 1 Institute of Basic Theory, China Academy of Chinese Medical Sciences, No.16 Nanxiaojie, Dongzhimennei, Beijing 100700, China; Email: zzgtcm@163.com; 2 Institute of Basic Research in Clinical Medicine, China Academy of Chinese Medical Sciences, No.16 Nanxiaojie, Dongzhimennei, Beijing 100700, China; Email: niuxuyan@yahoo.cn (X.N.); lv_cheng0816@163.com (C.L.); miao_jm@vip.126.com (M.J.); 3 Functional Genomics & Proteomics Laboratory, Osteoporosis Research Center, Creighton University Medical Center, 601N 30th ST, Suite 6730, Omaha, NE 68131, USA; 4 School of Chinese Medicine, Hong Kong Baptist University, 7 Baptist University Road, Kowloon Tong, Hong Kong, China

**Keywords:** curcumin, fine particulate matter, protein-protein interaction network, bioinformatics prediction, pathway analysis

## Abstract

Fine particulate matter (PM_2.5_) has been associated in humans with inflammation, oxidative stress and cancer. Studies had shown that curcumin could potentially inhibit these effects; however, there had been no *in vivo* or *in vitro* reports about the effects of curcumin on organisms exposed to PM_2.5_. This predictive study explored the possible biological functions and pathways involved in the mechanism of curcumin inhibition of the hazardous effects of PM_2.5_. For predictive analysis, microarray data were used to investigate the effect of PM_2.5_ on human bronchial epithelial cells (HBEC), and human target proteins of curcumin were retrieved from PubChem. Two protein-protein interaction (PPI) networks were established based upon differential genes and target proteins, respectively, and the common network of these two networks was found. Functional and pathway analysis of the common network was performed using the Ingenuity Pathways Analysis (IPA) software. The results suggested that the predictive effects of curcumin on HBEC exposed to PM_2.5_ were involved in bio-functions, including inflammatory response of airway, cancerogenesis, and apoptosis, and in pathways such as cancer, glucocorticoid receptor signaling, and NF-kappaB signaling. This study predicted for the first time that curcumin could be a potential therapeutic agent for protecting the human airway from the hazardous effects of PM_2.5_.

## 1. Introduction

Air pollution had long been considered a hazard to human health. Ambient airborne particulate matter (PM), an important environmental pollutant, had been associated with multiple cardiopulmonary diseases and cancers [1]. In the past few decades, many studies had highlighted the role of the size and surface area of PM in determining the potential to elicit inflammatory injury, oxidative damage, and other biological effects [2]. These effects were stronger for fine particles (diameter < 2.5 μm, known as PM_2.5_), because they could penetrate deeper into the airways of the respiratory tract and reach the alveoli, where 50% of the PM_2.5_ were retained in the lung parenchyma [3]. In recent years, the hazardous effects of PM_2.5_ had captured more and more public attention. However, do we have other methods to protect us from the hazardous of PM_2.5_ in addition to reducing the discharge of PM_2.5_ into the atmosphere? Furthermore, can certain food or herbal additives intake actively defend the body against the damaging effects of PM_2.5_? 

Curcumin, a yellow pigment extracted from the rhizome of the plant *Curcuma longa* (turmeric), had been widely used as a spice, food additive, and herbal medicine in Asia [4]. In recent years, extensive *in vitro* and *in vivo* studies had suggested that curcumin had anticancer, antiviral, antiarthritic, anti-amyloid, antioxidant, anti-inflammatory, and anti-aging properties [5]. Interestingly, these therapeutic effects of curcumin were in direct opposition to the detrimental effects of PM_2.5_. Therefore, we speculated that curcumin as a therapeutic agent might control or decrease the damage induced by PM_2.5_. In the present study, we predicted the underlying protective mechanism of curcumin on human airway epithelial cells (HBEC) exposed to PM_2.5_ based on gene expression profiling in Gene Expression Omnibus (GEO) and target protein data in PubChem.

## 2. Results and Discussion

### 2.1. Results

Using a t-test, we identified 89 genes differentially expressed between HBEC exposed to PM_2.5_ and vehicle control (Table 1). These genes could clearly distinguish primary HBEC exposed to PM_2.5_ from the HBEC in control. Of the 89 genes, 38 genes were significantly up-regulated and 51 genes were remarkably down-regulated.

**Table 1:** Differentially expressed genes in HBEC exposed to PM_2.5_ *versus* control.

Probe Set ID	RefSeq ID	Gene Symbol	*p*-value	Fold Change	Regulation
203665_at	NM_002133	*HMOX1*	0.0045	24.58	up
209921_at	NM_014331	*SLC7A11*	0.0004	10.45	up
202436_s_at	NM_000104	*CYP1B1*	0.0015	7.02	up
201266_at	NM_003330	*TXNRD1*	0.0014	6.96	up
205749_at	NM_000499	*CYP1A1*	0.0185	5.54	up
203925_at	NM_002061	*GCLM*	0.0006	3.71	up
202923_s_at	NM_001498	*GCLC*	0.0110	3.29	up
201468_s_at	NM_000903	*NQO1*	0.0139	2.92	up
204151_x_at	NM_001353	*AKR1C1*	0.0042	2.91	up
206172_at	NM_000640	*IL13RA2*	0.0110	2.72	up
211653_x_at	NM_001354	*AKR1C2*	0.0083	2.70	up
209387_s_at	NM_014220	*TM4SF1*	0.0472	2.47	up
210845_s_at	NM_002659	*PLAUR*	0.0104	2.18	up
206683_at	NM_003447	*ZNF165*	0.0090	2.14	up
212907_at	NM_021194	*SLC30A1*	0.0357	2.14	up
214211_at	NM_002032	*FTH1*	0.0270	2.08	up
208963_x_at	NM_013402	*FADS1*	0.0202	2.03	up
205767_at	NM_001432	*EREG*	0.0160	1.98	up
219475_at	NM_182981	*OSGIN1*	0.0103	1.98	up
207675_x_at	NM_057091	*ARTN*	0.0313	1.97	up
202842_s_at	NM_012328	*DNAJB9*	0.0309	1.96	up
202266_at	NM_016614	*TDP2*	0.0001	1.95	up
201625_s_at	NM_005542	*INSIG1*	0.0446	1.93	up
209882_at	NM_006912	*RIT1*	0.0114	1.93	up
201489_at	NM_005729	*PPIF*	0.0093	1.92	up
213112_s_at	NM_003900	*SQSTM1*	0.0191	1.91	up
204420_at	NM_005438	*FOSL1*	0.0323	1.80	up
202284_s_at	NM_000389	*CDKN1A*	0.0324	1.77	up
206907_at	NM_003811	*TNFSF9*	0.0032	1.74	up
219697_at	NM_006043	*HS3ST2*	0.0291	1.72	up
204970_s_at	NM_002359	*MAFG*	0.0032	1.69	up
213187_x_at	NM_000146	*FTL*	0.0471	1.68	up
212717_at	NM_014798	*PLEKHM1*	0.0319	1.66	up
206498_at	NM_000275	*OCA2*	0.0221	1.66	up
202672_s_at	NM_001674	*ATF3*	0.0041	1.57	up
202021_x_at	NM_005801	*EIF1*	0.0460	1.55	up
202067_s_at	NM_000527	*LDLR*	0.0128	1.54	up
204958_at	NM_004073	*PLK3*	0.0153	1.50	up
202207_at	NM_005737	*ARL4C*	0.0139	3.24	down
202887_s_at	NM_019058	*DDIT4*	0.0123	2.66	down
201890_at	NM_001034	*RRM2*	0.0097	2.26	down
211450_s_at	NM_000179	*MSH6*	0.0486	2.26	down
201849_at	NM_004052	*BNIP3*	0.0293	2.20	down
219250_s_at	NM_013281	*FLRT3*	0.0425	2.10	down
209120_at	NM_021005	*NR2F2*	0.0056	2.01	down
202464_s_at	NM_004566	*PFKFB3*	0.0289	1.99	down
208808_s_at	NM_002129	*HMGB2*	0.0462	1.99	down
203344_s_at	NM_002894	*RBBP8*	0.0280	1.97	down
218718_at	NM_016205	*PDGFC*	0.0044	1.97	down
207173_x_at	NM_001797	*CDH11*	0.0432	1.95	down
201669_s_at	NM_002356	*MARCKS*	0.0448	1.92	down
207826_s_at	NM_002167	*ID3*	0.0285	1.84	down
204967_at	NM_001649	*SHROOM2*	0.0141	1.80	down
202628_s_at	NM_000602	*SERPINE1*	0.0486	1.77	down
212599_at	NM_015570	*AUTS2*	0.0053	1.77	down
203274_at	NM_012151	*F8A1*	0.0118	1.76	down
208673_s_at	NM_003017	*SRSF3*	0.0188	1.76	down
203476_at	NM_006670	*TPBG*	0.0400	1.75	down
209189_at	NM_005252	*FOS*	0.0366	1.72	down
209784_s_at	NM_002226	*JAG2*	0.0032	1.70	down
203625_x_at	NM_005983	*SKP2*	0.0040	1.70	down
222036_s_at	NM_005914	*MCM4*	0.0439	1.66	down
202219_at	NM_005629	*SLC6A8*	0.0253	1.65	down
205449_at	NM_013299	*SAC3D1*	0.0328	1.65	down
212168_at	NM_006047	*RBM12*	0.0031	1.64	down
209286_at	NM_006449	*CDC42EP3*	0.0060	1.63	down
204334_at	NM_003709	*KLF7*	0.0105	1.63	down
208579_x_at	NM_017445	*H2BFS*	0.0173	1.62	down
204069_at	NM_002398	*MEIS1*	0.0281	1.60	down
203797_at	NM_003385	*VSNL1*	0.0172	1.58	down
203764_at	NM_014750	*DLGAP5*	0.0181	1.58	down
213051_at	NM_020119	*ZC3HAV1*	0.0104	1.58	down
208051_s_at	NM_006451	*PAIP1*	0.0321	1.57	down
203405_at	NM_003720	*PSMG1*	0.0304	1.57	down
211744_s_at	NM_001779	*CD58*	0.0273	1.57	down
206277_at	NM_002564	*P2RY2*	0.0179	1.56	down
204715_at	NM_015368	*PANX1*	0.0375	1.56	down
201312_s_at	NM_003022	*SH3BGRL*	0.0383	1.55	down
213088_s_at	NM_015190	*DNAJC9*	0.0253	1.55	down
203803_at	NM_016297	*PCYOX1*	0.0350	1.54	down
201624_at	NM_001349	*DARS*	0.0225	1.54	down
214214_s_at	NM_001212	*C1QBP*	0.0468	1.54	down
212320_at	NM_178014	*TUBB*	0.0185	1.53	down
208405_s_at	NM_006016	*CD164*	0.0465	1.51	down
213019_at	NM_012416	*RANBP6*	0.0002	1.51	down
212922_s_at	NM_020197	*SMYD2*	0.0002	1.50	down
209025_s_at	NM_006372	*SYNCRIP*	0.0481	1.50	down
201163_s_at	NM_001553	*IGFBP7*	0.0458	1.50	down
214800_x_at	NM_001207	*BTF3*	0.0036	1.50	down

57 human target proteins of curcumin (CID: 969516) were obtained from the PubChem database by PubChem Promiscuity online and identified by UniProt protein IDs (Table 2).

**Table 2:** Human target proteins of curcumin in PubChem.

GI	UniProtKB ID
4507949	1433B_HUMAN
31542303	ABHD5_HUMAN
37622910	ACM1_HUMAN
21361176	AL1A1_HUMAN
4885057	APJ_HUMAN
47132611	ATG4B_HUMAN
6683500	BAZ2B_HUMAN
53832009	CAC1H_HUMAN
4502601	CBR3_HUMAN
37187860	CCR6_HUMAN
67551261	CLK1_HUMAN
153791372	CLK3_HUMAN
13435386	CP3A4_HUMAN
32307159	CRFR2_HUMAN
30219	CRHBP_HUMAN
4503383	DRD1_HUMAN
4503385	DRD2_HUMAN
10835013	ESR2_HUMAN
4885263	GEM_HUMAN
122921310	HCD2_HUMAN
155969707	IDE_HUMAN
98986450	KC1G1_HUMAN
153791733	KC1G2_HUMAN
325651834	KCNH2_HUMAN
221046486	KD4DL_HUMAN
22035600	M4K2_HUMAN
11386165	MCL1_HUMAN
89993689	MDM2_HUMAN
88702791	MDM4_HUMAN
20986531	MK01_HUMAN
4505209	MMP13_HUMAN
66911845	MRGX1_HUMAN
34577122	NFKB1_HUMAN
222080095	OX1R_HUMAN
32307152	OXYR_HUMAN
4505587	PA1B3_HUMAN
5031975	PAK4_HUMAN
31881630	PE2R2_HUMAN
31542939	PGDH_HUMAN
4505811	PIM1_HUMAN
42821112	PIM2_HUMAN
223718196	PLIN1_HUMAN
116734717	PPBT_HUMAN
4826962	RAC3_HUMAN
41281453	SLK_HUMAN
23943882	STK33_HUMAN
8400711	TAU_HUMAN
223468676	TF65_HUMAN
4507533	TLR4_HUMAN
8394456	TLR9_HUMAN
4507615	TNNC1_HUMAN
151101270	TNNI3_HUMAN
48255881	TNNT2_HUMAN
4507681	TRFR_HUMAN
118600387	UBP1_HUMAN
4502331	V1AR_HUMAN
4507883	VDR_HUMAN

**Figure 1:**
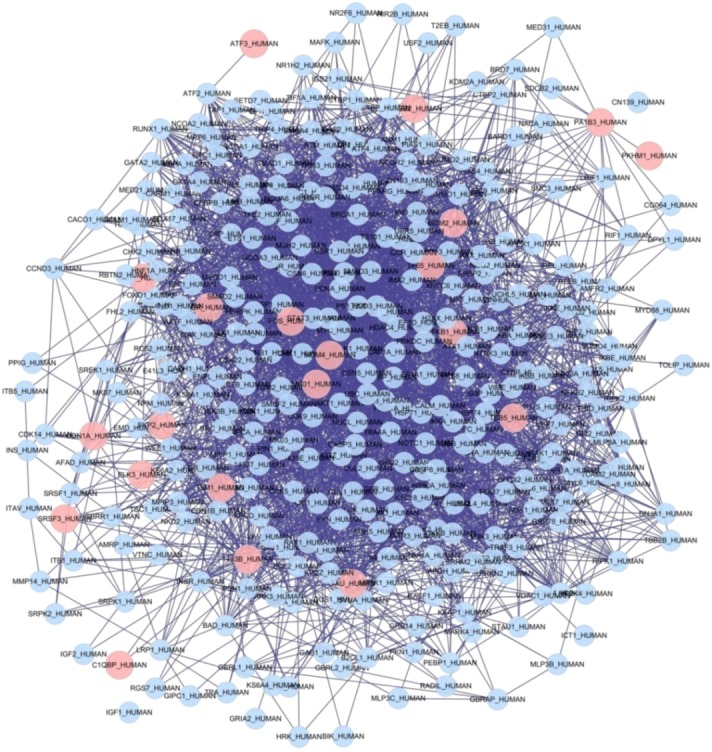
Common network of two PPI networks based on differentially expressed genes of HBEC exposed to PM_2.5_ and human target proteins of curcumin. Red cycles represent seed nodes, and blue cycles represent neighbor nodes. All edges represent interactions between the nodes.

Based on the differentially expressed genes in Table 1 and human target proteins in Table 2, two biological networks showing protein-protein interactions were constructed. The two protein-protein interaction (PPI) networks were visualized using Cytoscape. The nodes represented proteins in the PPI network and the edges represented the biological relationship between two nodes. There were 1,962 nodes and 15,455 edges in the PPI network of HBEC exposed to PM_2.5_ (Supplementary Material Figure S1), and 1,284 nodes and 11,541 edges in the PPI network of human target proteins of curcumin (Supplementary Material Figure S2). Appling the function “Intersection” of the Advanced Network Merge plugin in Cytoscape, we found the common proteins and relationships (common network) in the two PPI networks. The common network had 1,197 nodes and 9,521 edges (Figure 1).

The top five functions of the common network and the number of proteins associated with each function were found using Ingenuity Pathways Analysis (IPA). The most significant biological functions were grouped into three categories: (1) Diseases and Disorders, (2) Molecular and Cellular Functions, and (3) Physiological System Development and Function (Table 3).

**Figure 2:**
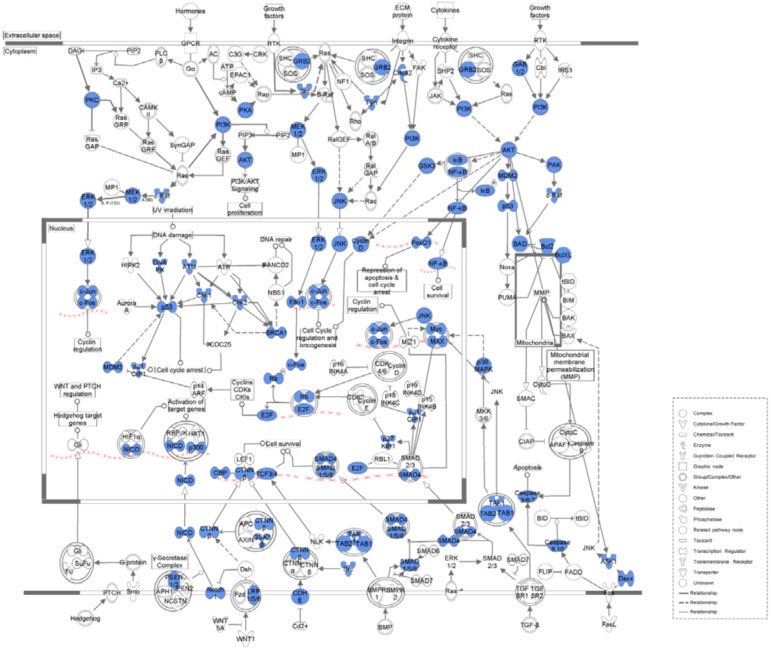
Molecular mechanisms of cancer associated with the common network. Blue legends represent proteins contained in the common network.

**Figure 3:**
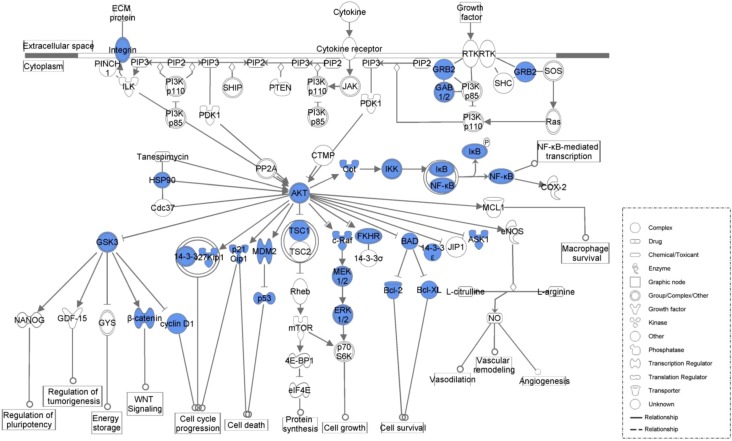
PI3K/AKT signaling associated with the common network. Blue legends represent proteins contained in the common network.

**Figure 4:**
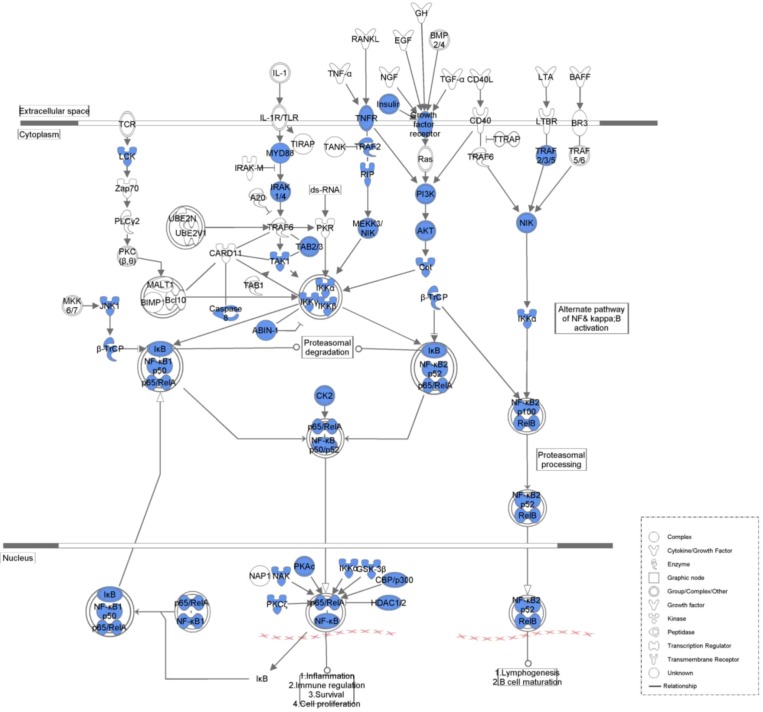
NF-kappaB signaling associated with the common network. Blue legends represent proteins contained in the common network.

**Figure 5:**
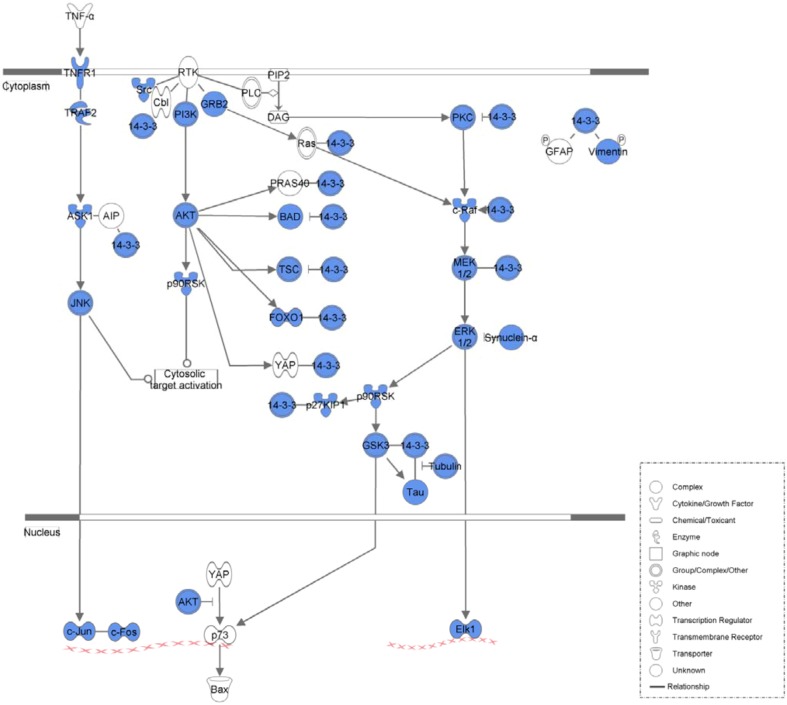
14-3-3-mediated signaling associated with the common network. Blue legends represent proteins contained in the common network.

Table 4 lists the top five canonical pathways associated with the common network as calculated by IPA (Figure 2, Figure 3, Figure 4 and Figure 5, Supplementary Material 3). Calculation was either according to ratio (the number of genes from the data set that map to the canonical pathway in question divided by the total number of proteins that map to the same canonical pathway) or significance (Fischer’s exact test was used to calculate a *P*-value determining the probability that the association between the proteins in the dataset and the canonical pathway was explained by chance alone).

**Figure 6:**
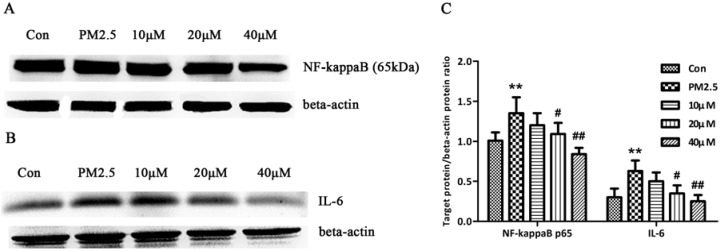
The effect of curcumin on the NF-kappaB p65 and IL-6 of 16HBE exposed to PM_2.5_. Cells were pre−treated with 10, 20, 40 μM curcumin for 30 min followed by exposure to PM_2.5_ (250 μg/mL) for 24 h in the presence or absence of curcumin. After 24 h, cells were collected and measured for NF-kappaB p65 and IL-6 expression by Western blot. (**A**) Expression of NF-kappaB p65. (**B**) Expression of IL-6. (**C**) Bar graphs showing the quantification of Western blot bands. Beta-actin was used as an internal control. ** *p* < 0.01, compared with the control group, ^#^ *p* < 0.05, ^##^ *p* < 0.01, compared with the PM_2.5_ group.

To partially validate the pathways listed in Table 4, we measured the expression of NF-kappaB p65 and IL-6 in human bronchial epithelial cells (16HBE) exposed to PM_2.5_. 16HBE were pre−treated with 10, 20, 40 μM curcumin for 30 min followed by exposure to PM_2.5_ (250 μg/mL) for 24 h in the presence or absence of curcumin. After 24 h, cells were collected and measured for NF-kappaB p65 and IL-6 expression by Western blot. Notably, NF-kappaB p65 or IL-6 expression level was markedly increased in 16HBE exposed to PM2.5 as compared with the control cells. However, curcumin treatment could attenuate the high expression of NF-kappaB p65 or IL-6 in cells induced by PM_2.5_ (Figure 6).

**Table 3:** Key functions associated with the common network using IPA.

Top Bio Functions	*p*-value	Number of Molecules
*Diseases and Disorders*		
Infectious Disease	1.26E−12–4.25E−02	35
Cancer	3.45E–3.01E−02	8
Genetic Disorder	1.33E–3.01E−02	5
Respiratory Disease	1.33E–3.01E−02	6
Inflammatory Response	2.79E–2.79E−02	1
*Molecular and Cellular Functions*		
Cell Death	9.91E−20–3.01E−02	31
Cellular Growth and Proliferation	5.64E−15–2.79E−02	32
Cellular Development	1.56E−08–2.79E−02	17
Cell Cycle	1.84E−07–2.79E−02	12
Cellular Movement	1.01E−04–2.79E−02	10
*Physiological System Development and Function*		
Organismal Survival	2.02E−03–2.02E−03	4
Respiratory System Development and Function	2.28E−03–2.28E−03	2
Tissue Development	2.28E−03–2.79E−02	2
Connective Tissue Development and Function	1.94E−02–1.94E−02	2
Tissue Morphology	2.79E−02–2.79E−02	1

**Table 4:** Key canonical pathways associated with the common network using IPA.

Canonical Pathways	*p*-value	Ratio
Glucocorticoid Receptor Signaling	2.57E−42	61/238 (0.256)
Molecular Mechanisms of Cancer	6.68E−39	65/314 (0.207)
PI3K/AKT Signaling	6.87E−36	41/110 (0.373)
NF-kappaB Signaling	1.33E−30	41/143 (0.287)
14-3-3-mediated Signaling	1.37E−30	36/102 (0.353)

### 2.2. Discussion

Predictive analysis was a general method for predicting the accuracy of quantitative experiments. The use of predictive analysis allowed the designer of an experiment to estimate the accuracy that should be obtained from the experiment before the experimental setup was finalized [6]. Until now, there had been no *in vivo* or *in vitro* reports about the effects of curcumin on organisms exposed to PM_2.5_; therefore, we collected limited data associated with PM_2.5_ or curcumin available from online databases such as GEO and PubChem. Because the aim of our study was to outline the potential biofunctions and pathways associated with the effect of curcumin on HBEC exposed to PM_2.5_ predictively, we did not restrict all data reanalyzed at identical molecular level.

PubChem [7] is a public repository for biological properties of small molecules hosted by the US National Institutes of Health (NIH). The PubChem BioAssay database contained biological test results for more than 700,000 compounds. From the PubChem BioAssay database, we could retrieve the target proteins of compounds [8]. In our study, 57 human target proteins of curcumin (CID: 969516) were obtained.

PPI were extremely important cellular events that affected many of the most important molecular processes in the cell, such as DNA replication. They formed the basis for many signal transduction pathways and transcriptional regulatory networks. The availability of complete and annotated genome sequences of several organisms had led to a paradigm shift from the study of individual proteins in a cell to proteome−wide analysis in an organism. The whole proteome analysis had illustrated that PPI affected cellular biological functions through many orchestrating networks such as metabolic, signaling and regulatory pathways in an organism [9].

Within the airway, the epithelium forms the mucosal immune barrier, the first structural cell defense against common environmental insults such as microorganisms and particulate matter. Hence, respiratory infectious diseases share similar pathologic processes such as the inflammatory response or oxidative stress with bronchial diseases induced by PM_2.5_ [10,11,12]. The inflammatory response was the main acute effect induced by PM_2.5_ in the respiratory tract, a target organ of PM_2.5_. *In vitro* studies had shown that airway epithelial cells responded to PM_2.5_ exposure by the release of inflammatory cytokines such as IL-1beta, TNF-alpha, and IL-6 [13], chemokines such as IL-8 [14], and erythropoietic cytokines such as G-CSF and GM-CSF [15,16]. Because curcumin was observed to inhibit secretion of the pro-inflammatory cytokines NF-kappaB mediating in HBEC exposed to pollutants [17,18], we predicted that curcumin might also have an anti-inflammatory effect on HBEC exposed to PM_2.5_. 

Some researchers conducting large epidemiological cohort studies in the United States and Europe had comfirmed the relationship between long-term exposure to particulate air pollution (PM_10_ and PM_2.5_) and increased mortality from lung cancer, especially in combination with other known risk factors, such as smoking, passive smoking, and occupational exposure [19,20]. By contrast, curcumin, a natural antitumor compound, had been shown to have the effect of inhibiting lung cancer cell invasion and metastasis in several studies [21,22,23] and have promising potential as a diet-derived cancer chemopreventive agent [24]. Thus, we inferred that curcumin could inhibit the carcinogenesis of airway epithelial cells resulting from PM_2.5_ exposure. 

Generally, PM_2.5_ led to the proliferation inhibition and apoptosis of HBEC [25,26,27]. PM_2.5_ could induce cell cycle arrest in G1 phase, inhibit DNA synthesis, and block airway epithelial cell proliferation [28]. The P53 pathway, tumor necrosis factor-alpha (TNF-alpha) pathway, and mitochondrial pathway played critical roles in the apoptosis processes induced by PM_2.5_ [29,30]. However, as a dietary antioxidant, curcumin had been proven to have preventive potential against apoptosis induced by peroxide or cigarette smoke extract in HBEC though inhibition of NF-kappaB [17,31]. Moreover, curcumin was a selective apoptosis modulator. For most noncancerous cells, curcumin was a protector and prevents cells from apoptosis induced by various adverse factors, but for cancer cells, curcumin was a killer and arrested cell cycle, inhibited cell proliferation, and/or caused apoptosis. For example, when mammary epithelial cells and breast cancer cells accumulated a similar amount of curcumin, a significantly higher percentage of apoptotic cells was induced in cancer cells compared to epithelial cells [32]. Similarly, we speculated that curcumin might have a two-way regulating effect on HBEC when exposed to PM_2.5_. 

Glucocorticoids (GCs) could control airway inflammation in respiratory diseases such as chronic obstructive pulmonary disease (COPD) and asthma, and the airway epithelium was a primary target of GC anti-inflammatory actions [33]. GC effects were mediated through the GC receptor (GR). Previous studies had indicated that cultured HBEC from smokers possess GRs with a lower binding affinity, and this might result from the inflammation found in the airways in smokers [34]. Although there had been no studies involving the effect of PM_2.5_ on GRs in HBEC, cigarette smoke and PM_2.5_ shared a similar inflammatory effect on HBEC, and we could speculate that PM_2.5_ might decrease GR signaling. In addition, GR action was shown to be tightly regulated by histone deacetylase 2 (HDAC2), which suppressed inflammatory gene expression in inflammatory airway disease [35]. Acting as an HDAC activator, curcumin was found to restore HDAC2 activity, thereby restoring the function of the GR [36]. In summary, regulation of the GR pathway was a possible mechanism by which curcumin inhibits the hazardous effects of PM_2.5_. 

Recent studies have suggested that numerous components of phosphoinositide 3-kinase (PI3K)-dependent signaling, mediated by Akt kinase, played a crucial role in the expression and activation of inflammatory mediators, inflammatory cell recruitment, immune cell function, airway remodeling, and corticosteroid insensitivity in chronic inflammatory respiratory disease [37], especially in COPD and asthma [38]. PM_2.5_ or cigarette smoke could induce activation of the PI3K/Akt pathway in HBEC and promote transcription of downstream inflammatory mediators [39,40,41]. However, studies had proved that curcumin could inhibit PI3K/Akt/NF-kappaB signals in human lung epithelial cells [42], block Akt translocation to the nucleus and further decrease inflammation in human tracheal smooth muscle cells [43]. Therefore, we predicted that curcumin also might have potential to prevent HBEC from the toxicity effects of PM_2.5_ by modulating PI3K/Akt signaling. 

Recent research indicated that the NF-kappaB/IkappaB pathway played an important role in the inflammatory response induced by PM_2.5_ in the lung [44]. The activation of the NF-kappaB/IkappaB complex preceded cytotoxicity or inflammation in PM_2.5_-exposed human bronchial or lung epithelial cells through the reactive oxygen species (ROS)-dependent NF-kappaB pathway [45,46]. As an inhibitor of NF-kappaB, curcumin exhibited a potent anti-inflammatory effect, and could decrease the airway epithelial cell inflammatory cytokine response to the pollutant cadmium or cigarette smoke extract [17,18]. Like cadmium and cigarette smoke, PM_2.5_ was also a pollutant in the environment, so we hypothesized that curcumin might perform its anti-inflammatory effect on PM_2.5_ by inhibiting the NF-kappaB pathway. 

14-3-3 family members tightly regulated cell fate through interaction with a wide spectrum of proteins that were targeted by various classes of protein kinases [47]. 14-3-3 proteins played particularly important roles in coordinating the progression of cells through the cell cycle, regulating their response to DNA damage and influencing life−death decisions [48]. Studies reported that 14-3-3 might contribute to lung tumorigenesis. In H322 cells, over-expression of 14-3-3 protein resulted in abnormal DNA replication and polyploidization [49], and in A549 cells, 14-3-3 promoted cellular proliferation [50]. Other studies found that curcumin could induce the typical features of apoptosis and inhibited the expression of 14-3-3 in HT-29 cells [51]. Based on the evidence mentioned above, we predicted that curcumin might prevent HBEC exposed to PM_2.5_ from carcinogenesis by inhibiting the 14-3-3 pathway.

In the NF-kappaB signaling pathway, NF-kappaB played a pivotal role as inflammatory response regulator, and IL-6 was an important inflammatory factor regulated by NF-kappaB and caused the damage response of PM_2.5_ [52]. Therefore, in a validating experiment, we selected NF-kappaB p65 and IL-6 as validated molecules and found that curcumin treatment could attenuate the high expression of NF-kappaB p65 or IL-6 in cells induced by PM_2.5_. These results supported our part prediction. 

## 3. Experimental

### 3.1. Microarray Data Analysis

A microarray dataset (accession number GSE7010) [53] was downloaded from the GEO [54], and analyzed it based on the Affymetrix Human Genome U133A Array. This dataset was derived from a study observing global gene expression in HBEC and identifying cellular pathways associated with coarse, fine and ultrafine particulate matter exposure. Ambient PM was collected in three different size fractions from Chapel Hill air; particles were extracted from foam or filter matrices and lyophilized. Primary HBEC were exposed to PM_2.5_ at 250 μg/mL or vehicle control for 6 h in culture [55]. In this study, we used three samples from the control group (GSM161787, GSM161793, GSM161798) and three samples from the fine particulate matter (PM_2.5_) group (GSM161790, GSM161796, GSM161801). Probes showing differential expression were extracted by volcano plot analysis with the filtering criteria of a 1.5-fold change using GeneSpring GX version 11.0 after per chip and per gene normalization. 

### 3.2. Target Proteins of Curcumin

The human target proteins of curcumin (CID: 969516) in PubChem [56] were retrieved using PubChem Promiscuity [57] online [58] with the filtering criteria of “not less than one Active Bioassay”.

### 3.3. Construction of PPI Networks and Detection of Common Network

PPI represented a basic blueprint for the analysis of self-organization and homeostasis in living organisms [59]. In this study, a Cytoscape [60] plugin, BisoGenet [61], was applied for assembling the PPI network. Information on human PPI networks involving relevant genes was obtained from various databases, including HPRD (Human Protein Reference Database), BIND (Biomolecular Interaction Network Database), BioGRID (The General Repository for Interaction Datasets), DIP (Database of Interacting Proteins), IntAct (Database system and analysis tools for protein interaction data), and MINT (Molecular Interactions Database). Two PPI networks were constructed based on the differential expression of genes from microarray data analysis and the target proteins of curcumin from PubChem. Another Cytoscape plugin, Advanced Network Merge, was used to find the common proteins and relations (common network) in the two PPI networks. 

### 3.4. Functional and Pathway Analysis of Common Network

For further analysis, a data file was uploaded into IPA (Ingenuity® Systems, www.ingenuity.com, Redwood City, CA, USA). This file contained the proteins in the common network. Each protein identifier was mapped to its corresponding protein object in the Ingenuity Pathways Knowledge Base (IPKB). The functional analysis identified the biological functions and/or diseases that were most significant to the data set. Proteins from the data set that met the *P*-value threshold of 0.05 (Fisher’s exact test) and were associated with biological functions and/or diseases in the IPKB were kept for analysis. Canonical pathway analysis identified the pathways most significant to the data set, based on two parameters: (1) a ratio of the number of proteins from the data set that map to the pathway divided by the total number of proteins that map to the canonical pathway and (2) a *p*-value calculated with Fisher’s exact test determining the probability that the association between the proteins in the dataset and the canonical pathway is explained by chance alone.

### 3.5. Validating Experiment

#### 3.5.1. Chemicals

All reagents used in this validating experiment including curcumin (purity: 70%) were purchased from Sigma (Sigma-Aldrich, St. Louis, MO, USA) unless specified.

#### 3.5.2. Cell Culture

Human bronchial epithelial cells 16HBE were purchased from American Type Culture Collection (ATCC, Manassas, VA). Cells were maintained at 37 °C and 5% CO_2_ in DMEM medium supplemented with 10% heat-inactivated fetal bovine serum, 10 U/mL of penicillin and 10 U/mL of streptomycin. 

#### 3.5.3. Preparation of Particles

Urban atmospheric PM_2.5_ was kindly provided by Prof. Xiaohong Zhao of College of Arts and Sciences of Beijing Union University. PM_2.5_ was collected on 150 mm diameter nitrocellulose filters (HAWP, Sartorius, La Fert’esous-Jouarre, France) with a high volume sampler machine (DA-80 Digitel, Cugy, Switzerland, flowrate: 30 m^3^/h) during the winter of 2008 on the roof of a five−story building in Xueyuan Road, Haidian District, Beijing. Particles were processed as previously described [55].

#### 3.5.4. Treatment of Cells with Curcumin and PM_2.5_

The cells were pretreated with curcumin (10, 20, 40 μM) for 30 min followed by exposure to PM_2.5_ (250 μg/mL) for 24 h in the presence or absence of curcumin. After 24 h, total cell lysates were prepared and 30 μg protein was subjected to sodium dodecyl sulfate polyacrylamide gel electrophoresis (SDS-PAGE), followed by immunoblot analysis.

#### 3.5.5. Western Blot

Rabbit polyclonal anti-NF-kappaB p65, anti-IL-6 antibody and mouse monoclonal anti-beta-actin antibody were purchased from Cell Signaling Technology, Abcam Inc. and Applygen Technologies Inc., respectively. Goat anti-rabbit horseradish peroxidase−conjugated immunoglobulin G (IgG-HRP; Santa Cruz Biotechnology) and goat anti-mouse IgG-HRP (Santa Cruz Biotechnology) were used as secondary antibodies for the rabbit and mouse primary antibodies, respectively. Western blot was performed following the standard protocol. Precision Plus Protein^TM^ Dual Color Standards (Bio-Rad Laboratories) and PageRuler^TM^ Plus Prestained Protein Ladder (Fermentas) were used as molecular weight markers. The immunoblot was finally visualized by exposure on film with ECL Plus Western Blotting Detection Reagents (Amersham & Pharmacia Biotech). Each experiment was independently repeated in triplicate.

## 4. Conclusions

In this study, we predicted for the first time that the anticancer and anti-inflammatory effects of curcumin might play a key role in protecting human airway from the hazardous effect of PM_2.5_. Curcumin had the potential to be an airway-protective agent against PM_2.5_. The current findings were based on bioinformatic studies and require further investigation to confirm.

## Supplementary Materials

Supplementary materials can be accessed at: http://www.mdpi.com/1420-3049/17/10/12406/s1.
