# Supplementary figures and images for: The Effect of Curcumin on Human Bronchial Epithelial Cells Exposed to Fine Particulate Matter: A Predictive Analysis
Source: Molecules. 2012 Oct 22;17(10):12406–26. doi: 10.3390/molecules171012406 (PMC6268531; doi:10.3390/molecules171012406)

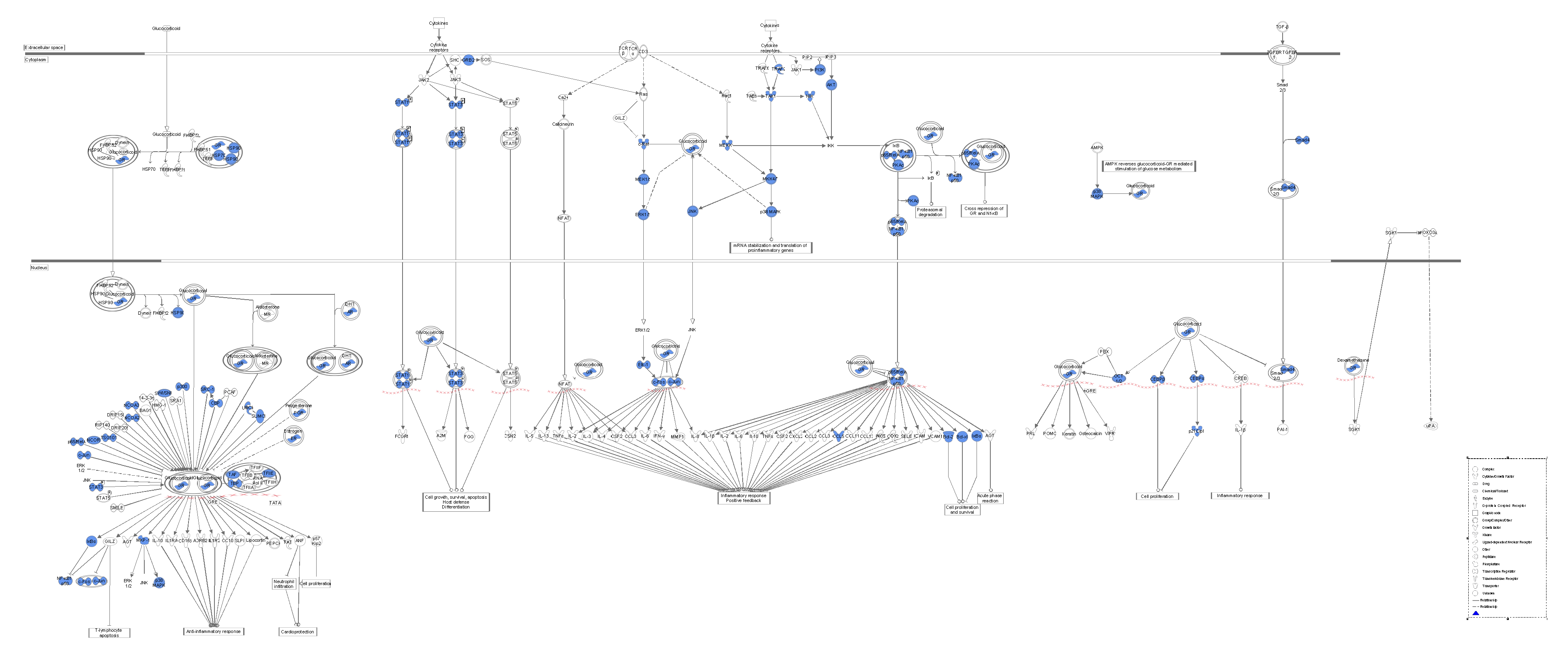

Supplement: Supplementary file 1 [file molecules-17-12406-s001.zip › supplementary material-3-Glucocorticoid Receptor Signaling.tif]
